# The Influence of Nanometals, Dispersed in the Electrophoretic Nanohydroxyapatite Coatings on the Ti13Zr13Nb Alloy, on Their Morphology and Mechanical Properties

**DOI:** 10.3390/ma14071638

**Published:** 2021-03-26

**Authors:** Michał Bartmański, Łukasz Pawłowski, Aleksandra Mielewczyk-Gryń, Gabriel Strugała, Krzysztof Rokosz, Sofia Gaiaschi, Patrick Chapon, Steinar Raaen, Andrzej Zieliński

**Affiliations:** 1 Faculty of Mechanical Engineering and Ship Technology, Gdańsk University of Technology, 80-233 Gdańsk, Poland; lukasz.pawlowski@pg.edu.pl (Ł.P.); gabriel.strugala@pg.edu.pl (G.S.); andrzej.zielinski@pg.edu.pl (A.Z.); 2 Advanced Materials Centre, Gdańsk University of Technology, 80-233 Gdańsk, Poland; alegryn@pg.edu.pl; 3 Institute of Nanotechnology and Materials Engineering, Faculty of Applied Physics and Mathematics, Gdańsk University of Technology, 80-233 Gdańsk, Poland; 4 Division of Surface Electrochemistry & Technology, Faculty of Mechanical Engineering, University of Technology, 57-620 Koszalin, Poland; rokosz@tu.koszalin.pl; 5 HORIBA FRANCE S. A. S., Boulevard Thomas Gobert—Passage Jobin Yvon, 91120 Palaiseau, France; sofia.gaiaschi@horiba.com (S.G.); patrick.chapon@horiba.com (P.C.); 6 Department of Physics, Norwegian University of Science and Technology, NO 7491 Trondheim, Norway; steinar.raaen@ntnu.no

**Keywords:** nanometals, nanohydroxyapatite coatings, nanoindentation, scratch test, adhesion

## Abstract

In this work, nanohydroxyapatite coatings with nanosilver and nanocopper have been fabricated and studied. The presented results concern coatings with a chemical composition that has never been proposed before. The present research aims to characterize the effects of nanosilver and nanocopper, dispersed in nanohydroxyapatite coatings and deposited on a new, non-toxic Ti13Zr13Nb alloy, on the physical and mechanical properties of coatings. The coatings were obtained by a one-stage electrophoretic process. The surface topography, and the chemical and phase compositions of coatings were examined with scanning electron microscopy, atomic force microscopy, X-ray diffractometry, glow discharge optical emission spectroscopy, and energy-dispersive X-ray spectroscopy. The mechanical properties of coatings were determined by nanoindentation tests, while coatings adhesion was determined by nanoscratch tests. The results demonstrate that copper addition increases the hardness and adhesion. The presence of nanosilver has no significant influence on the adhesion of coatings.

## 1. Introduction

Titanium and its alloys are, thanks to their appropriate mechanical properties, highly corrosion resistant and biocompatible; the most promising metallic biomaterials for long-term load-bearing implants. However, these materials have two major shortcomings: they are not bioactive without proper surface treatment, and demonstrate no antibacterial prevention [1,2].

Introducing bioactivity into titanium implants—assumed to consist of either enhanced phosphate deposition in simulated body fluids, a proliferation of osteoblasts, or the ability to provide a strong implant–bone connection—is mainly achieved by deposition of calcium phosphate coatings [3]. Usually, these coatings are either hydroxyapatite (HAp), or nanohydroxyapatite (nanoHAp) [4,5,6,7], or such phosphate coatings in which Ca was substituted by another element [8].

To ensure antibacterial properties, either surface grafting, surface nanostructurization, or coatings deposition is utilized [9]. Regarding implant coatings, silver/nanosilver [10,11,12,13,14] or copper/nanocopper [15,16,17,18] additives are commonly described and recommended. The bactericidal effect of these nanostructures, against both Gram-positive and Gram-negative bacteria, is attained by modifying cell permeability and causing bacterial cell dysfunction and death [19,20]. The bactericidal activity of both nanometals is determined by their size, shape, chemical state, and agglomeration rate [19,21,22].

Mechanical properties are among the fundamental determinants of long-lasting and load-bearing implants [23]. Considerable differences between the properties of human bone and implants have been proven to trigger a “shielding effect” and subsequently the risk of the implant loosening [24]. A noticeable drawback of bioceramic coatings (e.g., HAp, nanoHAp) deposited on metallic implants lies in their tendency to fracture, and poor adhesion to the substrate [25]. The modification of the chemical composition of the deposited coatings may be considered as one of the approaches to mitigate this problem.

Numerous attempts for this type of HAp coating doping have been reported. The doping elements, including Zn [26,27,28,29,30,31,32], Mg [10,33], Zn/Mg [34], Fe [35], Sr [36], Sr/Mn [37], Sr/Cu [16], La/Cu [15], Ce [38,39], Si [40,41] were proposed to increase the mechanical strength of HAp/nanoHAp coatings. Moreover, the additions to HAp of oxides like ZnO, SiO_2_, MgO and Ag_2_O [42,43], and TiO_2_ [44,45,46], carbon nanotubes (CNTs) [27,47,48], quercetin [49], and aptamer [50] were also investigated.

In contrary to antibacterial studies [51,52], the tests on mechanical properties of metal-doped HAp coatings are scarce. As reported [11,53], the deposition of the Ag-HAp coating obtained by Radio Frequency (RF) magnetron sputtering resulted in increased hardness and a decrease in Young’s modulus. In other research [54], the exponential relationship between increasing porosity and decreasing Young’s modulus was found. The influence of a grain shape [55] and the presence of amorphous HAp [53] were also considered. Limited tests revealed that Cu addition contributes to an improvement in the adhesion strength of HAp coatings [56,57]. The synergetic effect of both nanometals was rarely studied.

In the present research, the effects of nanoAg, nanoCu, and particularly both elements together on the nanoHAp coating properties, especially the mechanical properties, were investigated and discussed. Owing to the ability to obtain relatively thin films that adhere well to the substrate and possess superior properties [58,59] compared to coatings obtained by plasma spraying, electrophoretic deposition (EPD) was employed in the experiment. The EPD process parameters were adjusted based on previous studies for nanoHAp coatings containing nanoAg and nanoCu [18,60,61]. Using a variety of techniques, the microstructure, physical, and mechanical properties of such coatings on the surface of Ti13Zr13Nb alloy have been determined. The research problem was undertaken due to the limited studies focusing on the mechanical properties of nanoHAp coatings doped with nanometals, especially both types of nanoAg and nanoCu particles. The research has been particularly aimed at an assessment of mechanical properties of nanoHAp coatings doped with nanometals, strictly nanoAg and nanoCu particles. These nanometals are suitable for antibacterial prevention, but recognition of whether they may or may not influence the mechanical properties of coatings, and what are the mechanisms of such effects, has only scarcely been undertaken for these two metals, and never for them both together.

## 2. Materials and Methods

### 2.1. Preparation of Specimens

The Ti13Zr13Nb alloy (Xi’an SAITE Metal Materials Development Co., Ltd., Xi’an, China) with the composition listed in Table 1 was used as a substrate. Samples of 4 mm thickness and 15 mm in radius, cut from the rod, were ground with Silicon carbide (SiC) abrasive papers of 220, 500, 800, 1200, and 2000 µm (Struers Company, Krakow, Poland) on a grinding machine (Saphir 330, ATM GmbH, Mammenlzen, Germany) at 400 rpm. Prior to deposition, the specimens were cleaned with 2-propanol (99.7%, POCH, Gliwice, Poland) and then with demineralized water (II purity class acc. PN-EN ISO 3696:1999) obtained by a single distillation (HLP 5, HYDROLAB, Straszyn, Poland) in an ultrasonic bath (Sonic-3, Polsonic, Warszawa, Poland) for 60 min at room temperature. The last stage of sample preparation was their immersion in 25% v/v HNO_3_ for 10 min in room temperature to remove oxides from the surface, followed by re-cleaning with demineralized water in an ultrasonic bath (Sonic-3, Polsonic, Warszawa, Poland) for 15 min also at room temperature.

### 2.2. Electrophoretic Deposition

The suspensions for electrophoretic deposition of coatings were prepared by dispersing appropriate amounts of nanoHAp (average grain size 20 nm, 99% purity, MK Nano, Missisauga, ON, Canada), nanosilver (with an average particle size of 30 nm, Hongwu International Group Ltd., Guangzhou, China), and nanocopper (with an average particle size of 80 nm, Hongwu International Group Ltd., Guangzhou, China) powders in ethanol (99.8%, POCH, Gliwice, Poland) and mixing them in an ultrasonic bath (Sonic-3, Polsonic, Poland) at room temperature for 1 h. Table 2 summarizes the exact compositions of the prepared suspensions. The deposition parameters were selected on the basis of previous studies of nanohydroxyapatite coatings on the Ti13Zr13Nb alloy presented in [5]. The experimental set-up included the Ti13Zr13Nb alloy sample as a cathode and Pt as an anode, both connected to the direct current (DC) power supply (MCP/SPN110-01C, Shanghai MCP Corp., Shanghai, China). The distance between electrodes was about 10 mm. The EPD was conducted at a voltage value of 30 V, deposition time of 2 min, at room temperature. The coated specimens were dried in ambient air for 24 h at room temperature, then heat-treated in a vacuum furnace (PROTHERM PC442, Ankara, Turkey) for 120 min at 800 °C to increase the density of the coatings and the bonding between the coating and the Ti13Zr13Nb substrate. The heating rate of 200 °C/h starting from room temperature was applied. The cooling of the specimens was performed within the furnace.

### 2.3. Microstructure, Phase, and Chemical Examinations

The surface morphology of the coatings was observed by a scanning electron microscope (SEM; JEOL JSM-7800 F, Tokyo, Japan). For the cross-section examination of the coatings, the specimens were embedded in hot-melt resin (Resine Phenololique, Verte 602, Lam Plan, Gaillard, France) via a hot-melt press (Opal 410, ATM, Mammelzen, Germany) and subsequently ground in accordance with the procedure adopted for the Ti13Zr13Nb alloy substrate. The chemical composition of the coatings was investigated using the energy-dispersive X-ray spectroscopy (EDS; EDAX Inc., Mahwah, NJ, USA). The phase identification was performed by the X-ray diffraction spectrometer (XRD; Philips X′Pert Pro, Eindhoven, The Netherlands). The atomic force microscope (AFM; NaniteAFM, Nanosurf AG, Liestal, Switzerland) was applied to study the surface topography by the non-contact mode at 55 nN force and in the area of 80.4 × 80.4 μm.

The profile analysis of the depth distribution of the elements in the coatings was examined by glow discharge optical emission spectrometry (GDOES) with the Horiba Scientific GD Profiler 2 (HORIBA, Kyoto, Japan). Pulsed RF operation was used as it has a good sensitivity without inducing the heating of the samples. The studies were carried out at low pressure (700 Pa), the applied RF generator power was 40 W, pulse frequency was 3000 Hz, and there was a duty cycle of 0.25 (resulting in an average power of 10 W). The plasma gas was argon, and the anode diameter was 4 mm. The GDOES signals of calcium (423 nm), phosphorus (178 nm), oxygen (130 nm), nitrogen (149 nm), hydrogen (122 nm), titanium (365 nm), niobium (316 nm), zirconium (339 nm), copper (325 nm), and silver (328 nm), were measured.

### 2.4. Nanoindentation and Nanoscratch Tests

The nanomechanical and nanoscratch studies were conducted with the nanoindenter (NanoTest Vantage; Micro Materials, Wrexham, UK) equipment using a Berkovich indenter. The mean values of hardness (H) and reduced Young’s modulus (E) in nanoindentation tests, and critical load (beginning of delamination) and critical friction force in nanoscratch tests were calculated based on 110 independent experiments for each technique, relatively. The maximum force was 5 mN, the loading and unloading times were 20 s each, the dwell period at full load was 10 s, and a distance between the subsequent indents of 20 µm were set up in nanoindentation tests. The Oliver–Pharr method was used to calculate the contact area of indenter. The E value was calculated, assuming the Poisson’s ratio of 0.25 and 0.30 for uncoated, and nanoHAp coated Ti13Zr13Nb alloy, relatively. The nanoscratch tests were performed at the maximum load of 200 mN (for nanoHAp/nanoAg/nanoCu—400 mN) and loading rate of 1.3 mN/s at a distance of 500 µm (for nanoHAp/nanoAg/nanoCu—1000 µm). The nanoscratch test used a spherical diamond indenter with 5µm radius. The adhesion of the coating was determined as corresponding to the abrupt change in friction force.

### 2.5. Statistical Analysis

The collected data were statistically analyzed using a one-way analysis of variance (ANOVA). To evaluate the normal distribution of the collected data, the Kolmogorov–Smirnov test was employed. Statistical significance was set at *p* <0.05. All results were expressed as means ± standard deviation (SD).

## 3. Results

### 3.1. Morphology and Topography of Coatings

**Figure 1:**
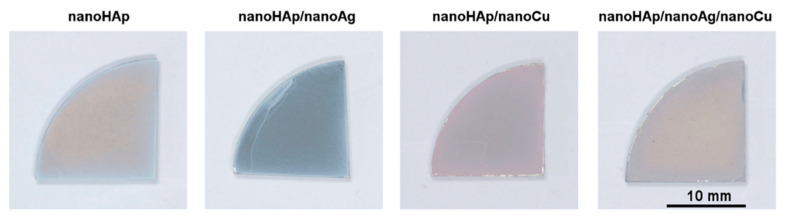
Nanohydroxyapatite-based coatings.

The macroscopic surface of nanohydroxyapatite-based coatings is presented in Figure 1. The color change of the surface of samples with nanoparticles compared to the nanoHAp coating suggests the presence of metallic nanoparticles inside the coating. The obtained coatings are homogeneous over the entire surface of the sample.

**Figure 2:**
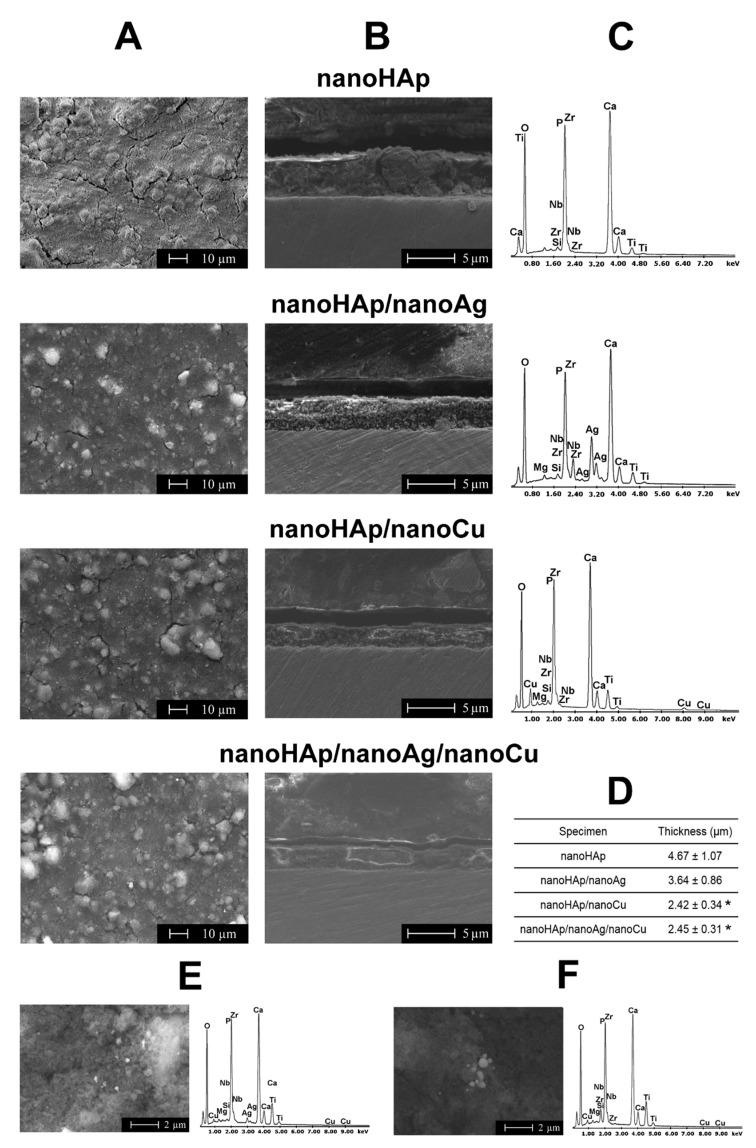
Scanning electron microscope (SEM) images of nanohydroxyapatite-based coatings. Coatings topography ((**A**) column), the thickness of the coatings (SEM images—(**B**) column, value—(**D**) table), elemental contents (**C**), and SEM images with energy-dispersive X-ray spectroscopy (EDS) spectra from the selected area. SEM images of topography and EDS spectra of nanoHAp/nanoAg/nanoCu coatings before (**E**) and after (**F**) heat treatment. (* significantly different results compared to the nanoHAp coating, according to a one-way analysis of variance (ANOVA) test followed by Tukey’s multiple comparison test, *p* <0.05).

The surface topography, thickness, and chemical composition of the investigated coatings are presented in Figure 2. Shallow cracks occurred, more and longer especially for pure nanoHAp coatings (column A). A significant amount of agglomerates appeared on nanoHAp and nanoHAp/nanoCu doped coatings, and for nanoHAp/nanoAg neither delamination, cracks, nor voids were observed (column B). The unevenness of the coatings was present; some surface irregularities of the coatings were also confirmed in roughness measurements by the AFM. For nanoHAp coatings doped with nanometals, a decreasing thickness was noticed (columns B and D). The EDS graphs (column C) confirmed the presence on the surface of three essential alloying elements, namely Ti, Zr, and Nb, and of the coating elements such as Ca, P, O, Ag, and Cu for two coatings. The EDS graphs for nanoHAp/nanoAg/nanoCu coatings are shown in columns E (before heat treatment) and F (after it). The presence of silver and copper was found in the analyzed area before heat treatment, while after treatment, no measurable amount of elemental silver was observed (columns E and F). Besides this, the distribution of elements both for the coating before and after heat treatment for such elements like oxygen, calcium, and phosphorus was uniform throughout the area. In the analyzed regions, metallic nanoparticles occurred mainly in the form of agglomerates.

**Figure 3:**
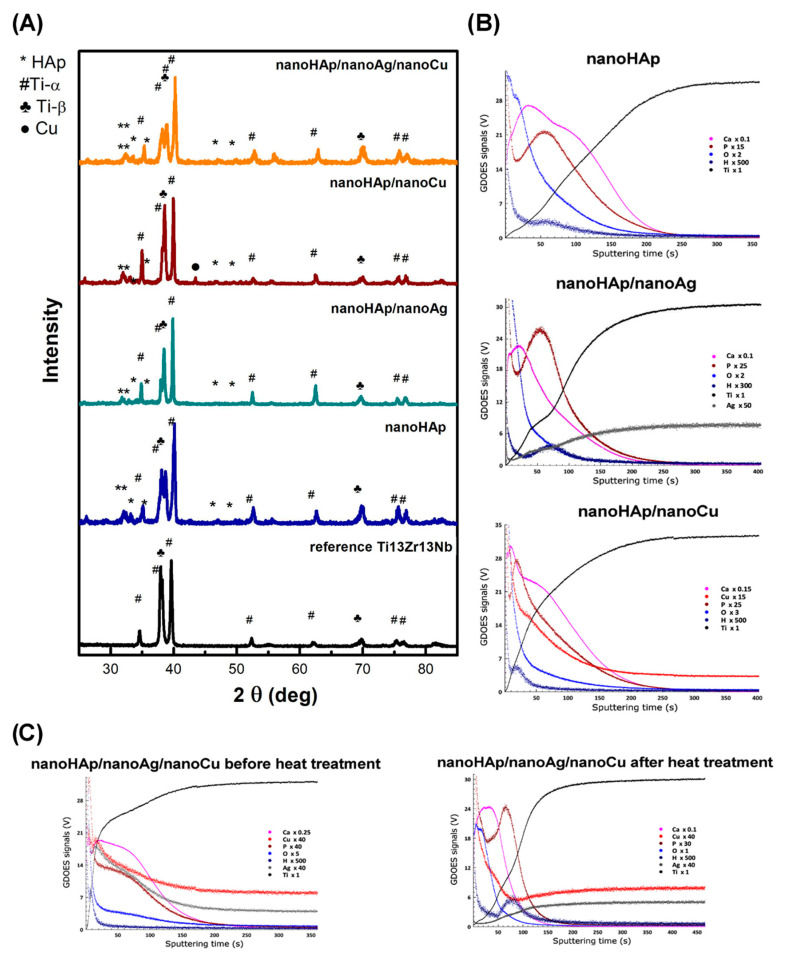
X-ray diffractograms of the reference Ti13Zr13Nb alloy and nanoHAp-based coatings (**A**) and glow discharge optical emission spectrometry (GDOES) graphs for tested coatings (**B**,**C**).

The results of X-ray analysis of the investigated samples are illustrated in Figure 3A. The main diffraction peaks originated from Ti. The different relative intensities of reflections indexed as coming from titanium α, and β polymorphs may be noticed. The diffraction peaks observed at indexes of 25.9°, 30.1°, 31.8°, 32.2°, 32.9°, 34.1°, and 39.8° are associated with HAp [62], those from Ag appear at 38.45°, 46.35°, 64.75°, and 78.05° [63], for Cu at 43.2°, 50.4°, and 74.1°, and nanoCu oxides at 36.4°, 42.3°, 61.3°, and 73.5° [64]. The low intensity of Ag in respect of reflections coming from HAp was previously noticed even for the bulk (not a layer) materials and for high Ag content [65]. This can suggest shallow content of nanoAg in the tested coatings. For nanoHAp/nanoCu coatings, the reflection associated with the nanoCu phase can be indexed.

The measured optical signals vs. sputtering time of nanoHAp-based coatings obtained with the GDOES technique are presented in Figure 3B,C. In the case of the nanoHAp reference sample (all specimens subject to the annealing), its top zone was enriched mainly in P and O with Ca depletion. That may be explained by the formation of substoichiometric nanoHAp with a Ca/P ratio lower than in the case of the bottom zone of the coating. The nanoHAp/nanoAg coating was only slightly enriched with Ag only in the top area. On the other hand, the copper presence in nanoHAp/nanoCu coating was the highest in the top layer and decreased over the entire thickness of that coating. The simultaneous addition of nanosilver and nanocopper (nanoHAp/nanoAg/nanoCu) made it possible to enrich the coating with both nanometals in its entire volume. However, after the heat treatment, the evaporation of Ag and enrichment of the top zone only in Cu was noticed.

**Figure 4:**
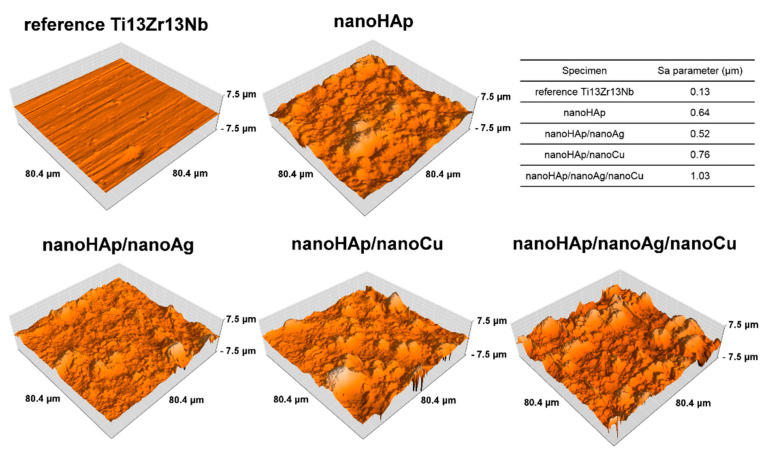
Atomic force microscope (AFM) topography of reference Ti13Zr13Nb alloy and nanoHAp-based coatings.

The AFM topographies of reference uncoated Ti13Zr13Nb alloy and nanoHAp-based coatings are presented in Figure 4. Three-dimensional topography maps of the bare titanium alloy sample revealed structure resulting from the grinding process. For the coated samples, the results indicated an agglomerated nanoHAp surface. The higher roughness was recorded for the coated samples in comparison with the reference sample. The AFM measurements correlated with the images shown in Figure 2, revealing the rough coating morphologies. The nanoHAp sample with the addition of both nanometals had the highest roughness.

### 3.2. Nanoindentation and Nanoscratch Tests

**Figure 5:**
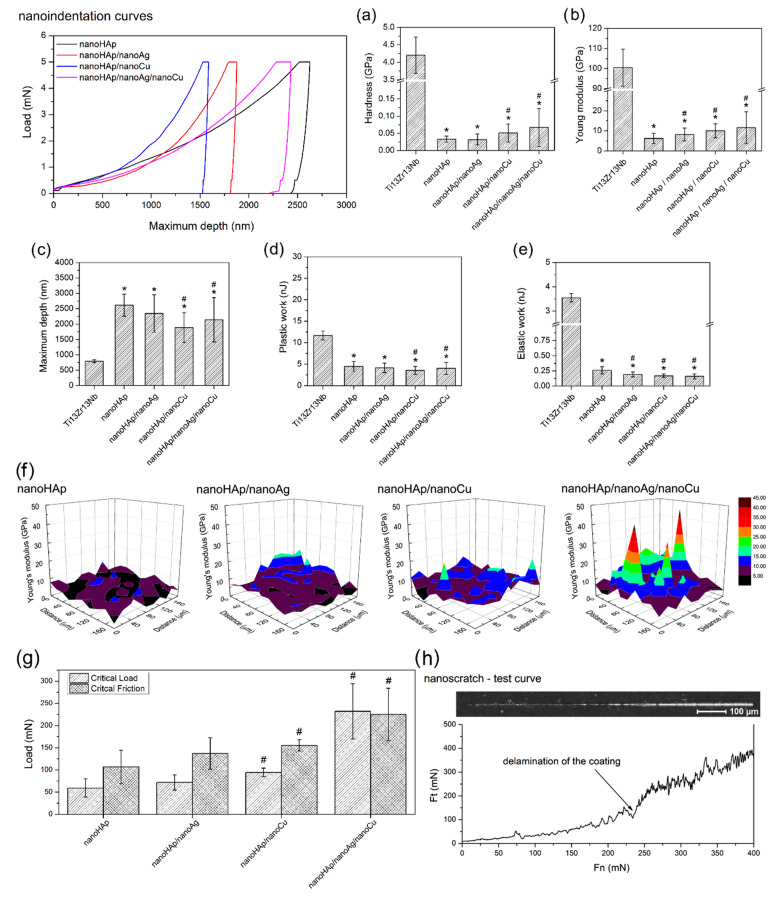
Nanoindentation curves and nanomechanical properties results for reference Ti13Zr13Nb alloy and nanoHAp-based coatings ((**a**)—hardness, (**b**)—Young’s modulus, (**c**)—maximum depth of indentation, (**d**)—plastic work, (**e**)—elastic work), maps the distribution of Young’s modulus for different coatings (**f**), nanoscratch test results (**g**), and nanoscratch test single curves for nanoHAp/nanoAg/nanoCu coatings (**h**) (* significantly different results compared to the reference Ti13Zr13Nb alloy, # significantly different results compared to the nanoHAp coating, according to one-way ANOVA test followed by Tukey’s multiple comparison test, *p* <0.05).

Nanoindentation curves, nanomechanical properties of reference Ti13Zr13Nb alloy and nanoHAp-based coatings, 3D Young’s modulus distribution, and nanoscratch test results for nanoHAp-based coatings are presented in Figure 5. The effect of the addition of Ag nanoparticles on the mechanical properties concerning the nanoHAp coating was negligible. An increase in hardness and Young’s modulus was evident for the nanoHAp/nanoCu coatings. The deposition of both nanometals improved the mechanical properties compared to the nanoHAp coating. At the same time, it did not cause a significant difference compared to the coatings doped with nanoAg. A significant maximum-depth deviation was reported for all measurements. The plastic work value exceeded the elastic work value for all samples. The amount of plastic work for all coatings increased as the maximum penetration depth of the indenter increased, and thus the hardness and Young’s modulus decreased.

In all tested coatings, in nanoscratch tests, an increase in the critical load value was found, along with the increasing critical friction force. The metallic nanoparticles addition into nanoHAp coatings increased the adhesion of these coatings in comparison with pure nanoHAp coating. In the case of nanoHAp coating with nanoAg and nanoCu, increasing critical load and friction force were observed.

## 4. Discussion

The addition of nanometals caused, for either of them and both together, a noticeable decrease in coating thickness, of 1.03 µm (nanoHAp/nanoAg), 2.22 µm (nanoHAp/nanoCu), and 2.25 µm (nanoHAp/nanoAg/nanoCu) when compared to nanoHAp coatings. The influence of nanometals on coating thickness was seldom investigated. In previous research [10], the addition of Ag lowered the coating thickness by about 6 µm. The decrease in thickness after adding Ag was explained by the slower deposition of ceramic particles in the presence of Ag particles [66]. The significant standard deviations of the coating thickness values were caused by high surface unevenness due to agglomerates of nanoHAp, nanoAg, and nanoCu particles.

The surfaces for all coatings demonstrated various, relatively low roughness. Increasing roughness was observed for all nanometals-doped coatings compared to the reference Ti13Zr13Nb alloy. The roughness increasing from an initial Sa = 0.13 µm to even 1.03 µm may be a valuable effect as the moderate roughness is essential for the adhesion of biomolecules resulting in appropriate primary stabilization of the implant in the bone [67,68]. The surfaces with a high roughness can enhance the production of the extracellular matrix [69,70]. On the other hand, roughness increase can also boost bacterial cell adhesion [70].

The effects of nanometals on the roughness of the HAp and nanoHAp coatings were thoroughly examined. No significant change in the surface roughness was found after the AgHAp (silver–hydroxyapatite) deposition by the RF magnetron sputtering [11]. An addition of copper to the SrHAp (strontium–hydroxyapatite) changed the surface from rough, needle-like, and flower-like to smoother and flake-like [16]. The increasing roughness of coatings with nanoAg and nanoCu may be understood as the hydroxyapatite compound and both metallic elements have different crystalline units and form interfaces, possessing high misfit.

The surface topography showed the aggregates of nanoHAp and metallic nanoparticles of copper. The aggregation (clustering) of nanometallic particles originates from the use of the EPD method involving suspensions of powders. The absence of the silver aggregated on the surface after heat treatment was confirmed by both GDOES and EDS results and can be explained as due to unexpected evaporation of nanoAg from the outer surface zone. This effect should not occur, as the melting temperatures of silver and copper are 961 °C and 1083 °C, respectively. However, for the nanoparticles, the melting and evaporation temperatures are not strictly defined. For the nanoAg particles of size 3.5 nm, the melting temperature was as low as 112 °C [71], and according to [72], the melting temperature ranged between 450 °C and 580 °C for Ag nanoparticles of sizes ranged from 4 to 20 nm. In the case of nanoCu, the melting temperature was reported as 180 °C for 35 nm and 580 °C for 62 nm of particle size [73].

The problem of silver evaporation is severe and a low melting point of nanometals has already been noted [74]. On the other hand, in [75], the powder AgHAp was deposited by electro-spraying and subject to the heat treatment at 500–900 °C for 2 h in air. Despite that, the ionic silver in HAp was still present. However, the most frequently Ag-substituted hydroxyapatites (not Ag in form of nanoparticles) obtained by the electrochemical deposition ECD method were investigated, and heat treatment of the coatings was not often imposed on what could negatively affect the mechanical properties of the coatings. Therefore, other deposition methods were attempted. For example, the microwave-irradiation and low processing temperature resulted in a uniform distribution of Ag^+^ ions within the apatite structure [13].

Despite that, the nanosilver evaporation was only partial. The presence of nanoAg inside the coatings is evident, as shown by the EDS and GDOES tests. The results mean that nanoAg, even after its possible partial evaporation from the surface, is still present at low content.

Taking into account the appearance of the negative phenomenon of nanoAg evaporation at elevated temperatures, we considered how to achieve its appropriate content within the coatings, and particularly on the surface. The first and often applied way is not to make any heat treatment of the phosphate coatings. Using this approach, there is no nanoAg loss, but the coatings remain soft, they contain non-crystalline Hap, and are less resistant to mechanical stresses. The adverse effect of heat treatment on nanoAg surface content may be minimized by a decrease in heat temperature and shortening of heating and cooling time, use of higher Cu nanoparticles, or use of only Cu-substituted HAp instead of Cu nanoparticles (even if the last are more efficient against bacteria).

The heat treatment of HAp coatings is often applied as it results in a phase transition, usually starting at 600 °C and followed by increasing hardness and adhesion of the coatings. In our research, the heating of coatings was carried out at 800 °C for 2 h. The formation of cracks in the coatings is due to thermal stresses [55], that occur during the annealing process of the samples. For the prepared samples, the cracks were relatively minor and short, hence their presence did not significantly affect the adhesion and hardness of the coatings. Utilizing the RF magnetron sputtering [11] and drying the coatings only in the air instead of heating [10], no surface cracking of the coatings was observed. On the other side, another important determinant of the cracking process is the retardation of nucleation and propagation of cracks, which are stopped by nanometals’ agglomerates. Regretfully, based on present results and taking into account only qualitative characterization of cracks, we are unable to ascertain which determinant among three considered is the most important, as they mostly affected porosity, thus decreasing the number of crack nuclei sites, or affected cohesion by stopping the cracks.

The effect of the presence of metal nanoparticles in the EPD suspension on the number and size of cracks in the coatings was noticed. The addition of nanoAg or nanoCu or both to the nanoHAp coatings reduced the number of cracks on the coating surface compared to the reference sample. This was most likely due to thermal stress relaxation by metallic agglomerates. The lowest number and length of cracks were found in the nano-HAp/nanoAg/nanoCu coating, which could be explained by the reduced porosity and increased cohesion of the coatings in the presence of soft metallic nanoparticles.

The damage of coating may manifest in two forms: (i) as a fracture of the coating under imposed stresses, i.e., cohesive failure, or (ii) as delamination at the titanium–coating interface, i.e., adhesive failure. Both phenomena, related to the presence of nanometals have not often been studied.

The adhesion is an essential property of HAp coatings, as the delaminated coating is unable to develop the bioactivity of an implant, and the particles of the coating may provoke local inflammation states and bone atrophy [76]. The adhesion is usually measured by the standard tapping method. The adhesion assessed by the glue method and under tensile stresses resulted in strength values such as 7.1–17.4 MPa for HAp [33,37,66,77], 14.5 MPa for SrMnHAp [37], 15.1 MPa for SrCuHAp [16], 15.9 MPa for AgHAp [66], and 16.7–18.1 MPa for MgHAp [77]. When the shear stresses were imposed, the adhesion strength achieved 11.7 MPa for HAp and 24.7–31.7 MPa for HAp/TiO_2_ coatings [78]. Recently, the nanoscratch process applied here has become popular as it makes it possible to assess the adhesion of a coating under shear stresses imposed. Such stresses may be considered as more likely to appear in daily human activity than tensile stresses often used in the tapping method. The previous nanoscratch tests brought out the critical load values 36–67 mN for nanoHAp and 40.6 mN for nanoHAp/nanoAg coatings [61], and in our other research, 109–124 mN for nanoHAp [79]. In the present tests, the critical load ranged between 60 and 210 mN, and the critical friction force between 110 and 220 mN. Similar values were found in [75] for friction forces, 60 to 350 mN, depending on heat temperature.

The addition of nanoAg, nanoCu, or both elements together increased the critical load, initiating the delamination of the coating. The opposite results were observed for the Ag addition [66] and explained by the higher porosity of such coatings. The increasing adhesion was attributed to rising grain boundary cohesion by reducing the grain border energy [16], or the formation of metallic bonds, or the toughening Cu effect preventing the crack propagation [56]. The last mechanism seems important for Ag and Cu nanoparticles in the studied coatings.

The mechanical properties of implant coatings are expected to be close to those of human cortical bone [24], and their chemical and crystalline forms decide on nucleation and dissolution rates in body fluids [80]. In previous research, the relatively high values reported for H and E for HAp coatings were 0.235 GPa and 5.6 GPa for HAp coating obtained by EPD on the 316 L steel [81]; 0.3 GPa and 45 GPa for laser-made layers [53]; 0.322–0.527 GPa and 10.8–26.8 GPa [82] on Mg alloy; 1 GPa and 6 GPa for HAp thin films obtained by sol-gel technique on the 316 L steel [24]; 5.4–153.5 and 5.2–19 GPa (before and after heat treatment) for EPD coatings on the Ti alloy [62]; 8 GPa and 140 GPa for bulk porous HAp [54], relatively. Here, the obtained results are relatively low as concerns hardness value, but the results obtained by the nanoindentation technique are highly sensitive to test parameters.

The performed tests show that nanoAg addition has negligible influence on the coating’s mechanical properties. Still, nanoCu presence, alone or with nanoAg, resulted in the increased value of hardness and Young’s modulus, i.e., made the coating more resistant to mechanical stresses. The addition of nanometals in our tests resulted in a change of hardness from 0.03 to 0.06 GPa, a change in Young’s modulus from 5 to 12 GPa, and a change in H^3^/E^2^ from 1.25 to 1.01 MPa, which could be assumed to be a substantial effect. The positive impact of nanoCu may be attributed to somewhat decreasing porosity and susceptibility to brittle cracking [56].

In order to obtain realistic values, it is necessary to have thicknesses of coatings apparently below the indent depth. In our experiments, the depth was even as great as 2.5 µm, close to the value of coating thickness. It means that the hardness response was for both coating and substrate. Therefore, we can only compare the differences between mechanical properties of various coatings. Nevertheless, the obtained results of hardness, Young‘s modulus, and nanoscratch responses, follow the changes in composition of coatings, such as an increase in all above values, even if they are not real, which is, however, typical for all measurements of thick layers and coatings.

## 5. Conclusions

This study presents the novel nanoHAp/nanoAg, nanoHAp/nanoCu, and nanoHAp/nanoAg/nanoCu composite coatings, obtained by electrophoretic deposition.

The one-step electrophoretic deposition of nanohydroxyapatite, nanosilver, or nanosilver and nanocopper together, on the surface of the Ti13Zr13Nb alloy, resulted in relatively thin composite coatings with specific morphology, surface topography, physical and mechanical properties.

The as-received composite coatings were characterized by high roughness and heterogeneity owing to the appearance of numerous aggregates of nanohydroxyapatite and nanometals.

Nanosilver and nanocopper increased adhesion and mechanical strength, presumably due to the increasing cohesion of grain boundaries.

The potentially antibacterial, biocompatible, well-adhered to the substrate, and mechanically strong coatings are developed for load-bearing titanium implants.

## Figures and Tables

**Table 1:** The chemical composition of the Ti13Nb13Zr alloy, wt.%.

Element	Zr	Nb	Fe	C	N	O	Ti
wt.%	13.0	13.0	0.05	0.04	0.019	0.11	rem.

**Table 2:** Test variables, investigated components, and their contents.

Specimen	Amount of nanoHAp (g/L)	Amount of nanoAg (g/L)	Amount of nanoCu (g/L)
nanoHAp	0.1	-	-
nanoHAp/nanoAg	0.1	0.01	-
nanoHAp/nanoCu	0.1	-	0.01
nanoHAp/nanoAg/nanoCu	0.1	0.005	0.005

## Data Availability

The data presented in this study are available on request from the corresponding author.
